# Associations of Dietary Factors, Body Mass Index, and Physical Activity with Tinnitus: A Scoping Review

**DOI:** 10.3390/jcm15114274

**Published:** 2026-06-01

**Authors:** Danuta Raj-Koziak, Szymon Chmiela, Henryk Skarżyński, Piotr H. Skarżyński

**Affiliations:** 1 Tinnitus Department, World Hearing Center, Institute of Physiology and Pathology of Hearing, 02-042 Kajetany, Poland; 2 Department of Otorhinolaryngology, Center of Hearing and Speech Medincus, 05-830 Kajetany, Poland; chmiela.szymon@gmail.com; 3 Interdisciplinary Student’s Scientific Society at the World Hearing Center, Institute of Physiology and Pathology of Hearing and Medical University of Warsaw, 02-091 Kajetany, Poland; 4 Faculty of Medicine, Medical University of Warsaw, 02-091 Warsaw, Poland; 5 Oto-Rhino-Laryngology Surgery Clinic, World Hearing Center, Institute of Physiology and Pathology of Hearing, 02-042 Kajetany, Poland; 6 Teleaudiology and Screening Department, World Hearing Center, Institute of Physiology and Pathology of Hearing, 02-042 Kajetany, Poland; 7 Institute of Sensory Organs, 05-830 Kajetany, Poland; 8 Heart Failure and Cardiac Rehabilitation Department, Faculty of Medicine, Medical University of Warsaw, 02-091 Warsaw, Poland

**Keywords:** tinnitus, diet, dietary interventions, physical activity, body mass index (BMI), obesity, quality of life

## Abstract

**Background**: Emerging evidence suggests that metabolic, nutritional, and lifestyle-related factors may be associated with tinnitus occurrence and symptom burden. Nutritional status, obesity, and sedentary behavior have been hypothesized to be linked with auditory function, neural excitability, and tinnitus-related outcomes. This scoping review aimed to map and summarize the available evidence on associations between dietary factors, nutrient intake, body mass index (BMI), obesity, physical activity, and tinnitus occurrence, severity, and related clinical outcomes. **Methods**: A scoping review was conducted in accordance with the PRISMA-ScR reporting guidelines. A comprehensive search of PubMed, Web of Science, and Cochrane Library databases was performed. Eligible designs included randomized controlled trials, cohort studies, case–control studies, and cross-sectional studies. Data were extracted and synthesized narratively due to methodological heterogeneity. **Results**: Twenty-four studies met the inclusion criteria. Several observational studies reported associations between protein intake, lipid profile, micronutrient status, BMI, obesity, physical activity, and tinnitus-related outcomes. Evidence on antioxidant supplementation was heterogeneous, with some trials reporting favorable changes in tinnitus-related measures and others showing no significant benefit compared with placebo. Elevated BMI, obesity, and altered body composition were generally associated with tinnitus occurrence or greater symptom severity. Randomized trials suggested that structured lifestyle programs involving dietary modification, weight reduction, and physical activity may be associated with improvements in tinnitus severity and quality of life in selected patient groups. **Conclusions**: The available literature suggests potential associations between nutritional, metabolic, and lifestyle-related factors and tinnitus occurrence or symptom severity. However, the evidence is heterogeneous and largely observational, with inconsistent adjustment for hearing loss, psychological distress, and general health status. Further well-designed prospective studies and randomized controlled trials are needed before causal or clinical recommendations can be formulated.

## 1. Introduction

Tinnitus is the subjective perception of sound without an external source [1]. This condition affects a significant portion of adults, and its prevalence increases with age. In Poland, the issue affects approximately 20% of adults and as much as 53% of those over 75 [2]. Moreover, a large-scale study involving 43,064 children in Warsaw revealed a notable underreporting of tinnitus in younger populations [3]. It is estimated that tinnitus occurs in 10–15% of adults in the United States, with around 20% of those suffering from the condition requiring medical intervention [1].

Tinnitus is not a disease, but rather a symptom that often takes a chronic form, significantly impacting a patient’s quality of life by causing sleep disturbances, concentration problems, and chronic levels of psychosomatic stress.

Despite the increasing prevalence of tinnitus and much research, the mechanism of tinnitus is still unclear [4]. Moreover, no unified diagnostic consensus has ever been established. Tinnitus can result from various causes, including hearing damage, neurological disorders, genetic causes [5], noise exposure [6], and vascular and somatic factors such as temporomandibular joint dysfunction [1]. Some studies also suggest a potential link between tinnitus and inflammation [7], patient’s dietary patterns, nutrient intake, BMI and weight reduction strategies [8]. Alcohol consumption has also been considered a potential lifestyle-related factor that may aggravate tinnitus perception in some patients or act as a confounding variable through its associations with sleep quality, psychological distress, vascular function, and general health status.

To date, studies have not provided conclusive evidence for the existence of a therapy that eliminates tinnitus. Modern therapeutic strategies focus mainly on symptom relief and improving the patient’s quality of life. There are many different questionnaires or tools to assess tinnitus [9,10,11]. Interest has been given to methods supporting habituation or techniques of managing distress caused by chronic tinnitus. High-level evidence demonstrates the efficacy and safety of Cognitive Behavioral Therapy (CBT) in the management of tinnitus. This conclusion is supported by systematic reviews and corroborated by randomized controlled trials, establishing CBT as the most robustly validated intervention for this condition [12]. Conversely, current research does not provide evidence supporting the efficacy of pharmacological interventions specifically for tinnitus. Moreover, available data indicate a risk of potentially significant adverse effects, thereby limiting the role of drug therapy in routine clinical practice. Tinnitus frequently coexists with hearing impairment, which may influence both symptom perception and treatment strategies. Hearing aids are strongly recommended in cases of concurrent hearing impairment. In addition to facilitating auditory rehabilitation, they should be considered a viable therapeutic option for patients experiencing both tinnitus and hearing loss. Cochlear implantation is reserved exclusively for patients who meet the established candidacy criteria related to the degree of hearing loss.

In addition to behavioral and sound-based approaches, several nutritional and metabolic interventions have been explored as potential strategies for tinnitus management.

The objectives of this study were as follows:

1. To perform a scoping review of all relevant studies investigating the role of diet, micronutrients, macronutrients, body mass index (BMI), obesity, weight loss, and physical activity in tinnitus occurrence, onset, severity, and tinnitus-related outcomes.
2. To include randomized controlled trials, cohort studies, case–control studies, and cross-sectional studies and to summarize their findings through synthesis presented in narrative analysis.

## 2. Materials and Methods

This study was conducted as a scoping review and reported according to the PRISMA-ScR guidelines (Preferred Reporting Items for Systematic Reviews and Meta-Analyses extension for Scoping Reviews) [13]. No protocol for this review was registered. A scoping review methodology was selected due to the heterogeneity of study designs, exposures, and outcome measures across the available literature. A comprehensive structured search was conducted across three major databases: PubMed, Web of Science, and the Cochrane Library. The search was carried out in September 2025 and covered the period from 2005 to 2025. The lower time limit of 2005 was chosen to ensure consistency with contemporary diagnostic criteria for tinnitus and with modern definitions of overweight and obesity, as well as to capture studies conducted after the widespread adoption of standardized instruments such as the Tinnitus Handicap Inventory (THI) and Visual Analogue Scale (VAS). Search terms were organized into four main concepts: tinnitus, diet and nutrition, BMI and obesity and physical activity. The same Boolean search strategy was used across all three databases. The core search string was: (tinnitus) AND (diet OR nutrition OR antioxidants OR vitamins OR minerals OR macronutrients OR micronutrients OR BMI OR body mass index OR obesity OR weight loss OR weight reduction OR physical activity OR exercise). To improve reproducibility, the database-specific implementation of the core search strategy for PubMed, Web of Science, and the Cochrane Library is provided in Supplementary Table S1. This strategy was intentionally broad and was designed to maximize sensitivity rather than specificity, in accordance with the aims of a scoping review. No additional disease-specific or metabolic terms were added to the original search string because the objective of the review was to map the available literature on tinnitus in relation to nutrition, body weight, and physical activity rather than to conduct a condition-specific review of metabolic syndrome or lipid disorders. Potentially relevant metabolic outcomes, including cholesterol, triglycerides, dyslipidemia, metabolic syndrome, waist circumference, and body composition, were considered during full-text screening and data extraction when they were reported within studies retrieved by the original search strategy. Exclusion criteria were defined a priori to ensure methodological rigor and consistency. We excluded editorials, letters, and conference abstracts without full data; non-human or in vitro studies; and studies without an available English full text. Articles were also excluded if they did not assess tinnitus as a primary outcome, did not investigate relevant exposures (dietary factors, micronutrients, macronutrients, BMI, obesity, weight loss, or physical activity), or provided insufficient data for extraction. Study selection was performed independently by two reviewers. In the first stage, titles and abstracts were screened in a blinded manner against the predefined eligibility criteria. In the second stage, potentially relevant articles were assessed in full text by the same two reviewers, also independently and in a blinded manner. Disagreements at both stages were resolved through discussion and consensus; if consensus could not be reached, an additional author was consulted. Data extraction was performed independently by two reviewers using a standardized extraction form developed before the extraction process. Extracted data were subsequently compared between reviewers, and discrepancies were resolved through consensus. Although no formal risk-of-bias assessment was performed, in line with the scoping-review design, key methodological limitations of each included study were systematically extracted and reported in Table 1 to support cautious interpretation of the evidence. Based on the search strategy, a total of 2107 records were identified. After removing duplicates, 1511 unique records remained. During the title and abstract screening phase, 1433 articles were excluded for not meeting the inclusion criteria. We assessed 78 articles for full-text eligibility, and after applying all exclusion criteria, 21 articles were left for qualitative synthesis. In addition to the primary search results, 3 relevant studies were identified through a review of the reference lists of included articles. Finally, a total of 24 studies were incorporated into the analysis.

**Figure 1:**
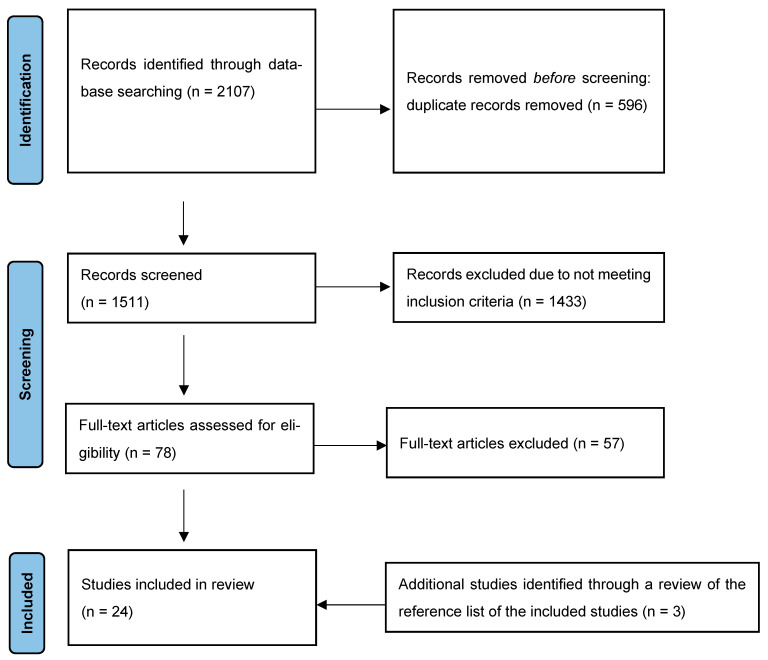
PRISMA 2020 Flow Diagram.

The study selection process is presented in a PRISMA flow diagram in Figure 1.

We included randomized controlled trials (RCTs), cohort studies, case–control studies, and cross-sectional studies that investigated dietary factors, body mass index (BMI), obesity, weight loss, or physical activity in relation to tinnitus.

Given the heterogeneity of study designs, populations, exposures, and outcome measures, we conducted a qualitative synthesis of the available evidence. Findings were synthesized narratively and organized thematically according to major exposure domains identified in the literature.

## 3. Results

The characteristics and main findings of all included studies are summarized in Table 1. The results are presented below in a narrative format, organized according to the major exposure domains.

### 3.1. Associations Between Macronutrient Intake and Tinnitus

#### 3.1.1. Protein Intake

In relation to protein intake, two observational studies reported inverse associations between higher protein consumption and tinnitus-related outcomes. In a large-scale, population-based study using UK Biobank data, Dawes et al. [14] examined dietary patterns and tinnitus symptoms among 34,576 participants. Dietary intake was assessed using the Oxford Web-Q, a computerized 24 h dietary questionnaire that captures intake of commonly consumed foods and beverages and enables estimation of macronutrient intake. After adjustment for demographic, lifestyle, and health-related factors, higher protein intake was associated with lower odds of reporting tinnitus (OR = 0.90; 95% CI, *p* < 0.05). Similar findings were reported by Jarach et al. [15] in a hospital-based case–control study including 185 patients with idiopathic tinnitus and 198 controls without tinnitus. Dietary habits over the previous year were assessed using a 37-item food frequency questionnaire covering cereals, protein-rich foods, fat-rich foods, vegetables, and fruits. The study found a statistically significant inverse association between consumption of selected protein-rich foods and tinnitus onset. Consuming three or more portions of poultry per week was associated with lower odds of tinnitus onset compared with zero or one portion per week (OR = 0.43; 95% CI, 0.23–0.81; *p* for trend = 0.009). Similar inverse associations were observed for prosciutto and legumes. Consumption of at least two portions of prosciutto per week was associated with lower odds of tinnitus (OR = 0.44; 95% CI, 0.23–0.85; *p* for trend = 0.019), as was consumption of at least two portions of legumes per week (OR = 0.50; 95% CI, 0.28–0.92; *p* for trend = 0.023).

#### 3.1.2. Fat Intake and Cholesterol Levels

Serum lipid levels and lipid-lowering interventions were examined in two studies with different designs. In a large-scale cross-sectional analysis of 6021 Korean adults aged 60 years or older from the 2016–2018 Korea National Health and Nutrition Examination Survey, Lee HJ et al. [16] investigated the association between serum lipid profile and tinnitus prevalence. Tinnitus was assessed through standardized health interviews and categorized by severity as “not annoying,” “irritating,” or “severely annoying and causing sleep problems.” Serum total cholesterol, triglycerides, LDL-C, and HDL-C were measured, with hypertriglyceridemia defined as triglycerides ≥ 200 mg/dL and a high TC/HDL-C ratio defined as >5.0. After adjustment for age, sex, hypertension, diabetes, dyslipidemia, smoking status, obesity, noise exposure, and psychological factors, hypertriglyceridemia was associated with higher odds of tinnitus (OR = 1.27; 95% CI, 1.04–1.56; *p* = 0.022), as was a high TC/HDL-C ratio (OR = 1.21; 95% CI, 1.02–1.44; *p* = 0.025). In severity analyses, both hypertriglyceridemia and a high TC/HDL-C ratio were also associated with higher odds of reporting “severely annoying” tinnitus. Sutbas et al. [17] evaluated changes in tinnitus severity in 42 male patients with hyperlipidemia undergoing a low-cholesterol diet or antihyperlipidemic therapy with simvastatin or atorvastatin for up to 24 months. Patients were classified as metabolically responsive if cholesterol or triglyceride levels normalized or decreased by at least 40% from baseline. In the responsive group (*n* = 20), tinnitus scores decreased from a mean of 4.95 ± 1.9 before therapy to 3.45 ± 2.6 after therapy (*p* < 0.05). Symptom ratings also differed between response groups: among responsive patients, 35% reported decreased tinnitus and 20% reported no tinnitus after therapy, whereas among non-responsive patients, 9% reported decreased tinnitus and 1 patient reported no tinnitus. These findings suggest that improvement in lipid parameters may be associated with more favorable tinnitus-related outcomes, although the study design limits causal interpretation.

### 3.2. Associations Between Micronutrient Status, Supplementation, and Tinnitus

#### 3.2.1. Vitamin B2, B3 and B12

Evidence concerning B-complex vitamins was reported in one population-based nutritional analysis and two clinical studies focused on vitamin B12 status or supplementation. Lee DY et al. [18] analyzed data from the sixth Korea National Health and Nutrition Examination Survey, including 7621 participants, of whom 1435 reported tinnitus symptoms and 6186 did not. Dietary intake was assessed using a food-frequency questionnaire covering 112 commonly consumed foods. In multivariate analyses, lower vitamin B2 intake was independently associated with higher tinnitus prevalence across all age groups (OR = 1.253; 95% CI, 1.049–1.496; *p* = 0.013). Age-stratified analyses showed significantly lower vitamin B2 intake among participants with tinnitus in the 51–55 and 56–60-year age groups. For vitamin B3, lower intake was correlated with greater tinnitus-related annoyance, reaching statistical significance in the 66–70 and 76–80-year age groups. Two clinical studies examined vitamin B12 status in relation to tinnitus. Berkiten et al. [19] assessed 100 patients with non-pulsatile tinnitus and 20 healthy controls, defining vitamin B12 deficiency as serum concentrations < 180 pg/mL. Vitamin B12 deficiency was common in both groups and did not differ significantly between tinnitus patients and controls (63% vs. 60%; *p* = 0.80; OR = 1.13). Among 63 deficient tinnitus patients who received parenteral B12 replacement for one year, VAS scores showed only a minimal, non-significant decrease from 6.40 ± 1.58 to 6.24 ± 3.05, although 8 patients reported subjective relief. In contrast, Singh C et al. [20] conducted a randomized, placebo-controlled trial involving 40 patients with chronic tinnitus, 42.5% of whom were vitamin B12-deficient. Participants received either intramuscular methylcobalamin or placebo for six weeks. Among deficient patients in the treatment group, significant reductions in tinnitus severity scores were observed, with TSI scores decreasing from 36.5 ± 8.2 to 28.2 ± 7.5 and VAS loudness decreasing from 6.5 ± 1.2 to 4.8 ± 1.1 (both *p* < 0.05). No comparable improvements were observed in non-deficient patients or in the placebo arm.

#### 3.2.2. Vitamin D3

Vitamin D status was examined in both clinical and population-based settings. In a case–control study, Nowaczewska et al. [21] compared serum 25-hydroxyvitamin D [25(OH)D] concentrations between 201 patients with chronic subjective tinnitus and 99 healthy controls. Mean vitamin D levels were significantly lower in tinnitus patients than in controls (19.86 ± 10.5 ng/mL vs. 27.43 ± 12.5 ng/mL; *p* < 0.0001), and vitamin D deficiency, defined as <20 ng/mL, was more frequent among tinnitus patients (50.7% vs. 22.2%; *p* < 0.0001). Within the tinnitus group, severe deficiency (≤15 ng/mL) was associated with higher THI scores (41.6 ± 20.1 vs. 32.4 ± 18.9; *p* < 0.05) and higher VAS loudness ratings (6.4 ± 1.5 vs. 5.3 ± 1.7; *p* < 0.05) compared with patients with vitamin D levels >15 ng/mL. A similar association was reported by Aliyeva et al. [22] in a nationally representative Korean National Health and Nutrition Examination Survey analysis including 16,408 adults aged 20 years or older. Vitamin D status was assessed using serum 25(OH)D concentrations categorized into quartiles. After adjustment for demographic and lifestyle factors, participants in the lowest vitamin D quartile had a higher prevalence of tinnitus than those in the highest quartile (adjusted OR = 1.24; 95% CI, 1.05–1.46; *p* = 0.012).

#### 3.2.3. Antioxidants and Multivitamin Supplements

Three studies evaluated antioxidant supplementation in relation to tinnitus outcomes, with heterogeneous findings across interventions and study designs. Petridou et al. [23] conducted a double-blind, placebo-controlled clinical trial in which patients with tinnitus were randomized to receive either antioxidant supplementation or placebo for three months. The antioxidant regimen included a multivitamin-multimineral preparation combined with α-lipoic acid. Of the 70 initially eligible patients, 63 completed the study, including 34 in the antioxidant group and 29 in the placebo group. After the intervention, the antioxidant group showed a significant reduction in tinnitus loudness, from 45.0 ± 15.3 dB to 30.8 ± 11.2 dB, corresponding to a mean change of −14.2 ± 12.7 dB (*p* < 0.001), whereas the placebo group showed a non-significant change from 47.1 ± 20.5 dB to 40.4 ± 15.5 dB (mean change −6.7 ± 8.8 dB; *p* = 0.168). Patient-reported outcomes also showed more favorable changes in the antioxidant group. THI scores decreased from 31.6 ± 19.3 to 25.5 ± 18.0 in the antioxidant group, while the placebo group showed a non-significant increase from 40.6 ± 27.7 to 42.8 ± 24.5. Interaction analyses indicated significant between-group differences, particularly for THI. Savastano et al. [24] examined 31 patients with unilateral idiopathic tinnitus who received antioxidant therapy for six weeks. The intervention included glycerophosphorylcholine, glycerophosphorylethanolamine, beta-carotene, vitamin C, and vitamin E. Oxidative stress was assessed using malondialdehyde (MDA) and 4-hydroxynonenal (4-HNE), measured in jugular venous blood based on prior evidence that oxidative stress markers may be detectable in cerebral veins in tinnitus patients [38]. After treatment, both oxidative stress markers decreased significantly, with MDA declining from 2.10 to 1.98 µmol/dL (*p* = 0.003) and 4-HNE from 2.36 to 2.16 µmol/dL (*p* = 0.002). Tinnitus-related outcomes also showed favorable changes, including a reduction in tinnitus loudness from 23.6 dB to 16.7 dB (*p* = 0.002) and a decrease in VAS-rated subjective disturbance from 58.0 to 35.7 (*p* = 0.001). No significant differences in clinical outcomes were observed between male and female patients. In contrast, Polanski et al. [25] reported no significant benefit of antioxidant regimens in a prospective, randomized, double-blind, placebo-controlled trial involving 58 elderly patients with chronic tinnitus associated with sensorineural hearing loss. Participants were assigned to receive Ginkgo biloba dry extract, α-lipoic acid with vitamin C, papaverine hydrochloride with vitamin E, or placebo for six months. Tinnitus outcomes were assessed using the Tinnitus Handicap Inventory. Across treatment groups, no statistically significant improvements were observed in THI severity grades or mean THI scores, either within groups or between groups. The distribution of THI severity grades remained unchanged (*p* = 0.441), and mean THI scores showed no significant reduction (*p* = 0.848). Subgroup analyses also failed to identify a treatment-related benefit.

#### 3.2.4. Minerals

Evidence concerning trace elements focused primarily on zinc and iron. Tang et al. [26] examined the association between dietary mineral intake and incident tinnitus in a 10-year prospective cohort study including 2947 adults aged 50 years or older. After baseline assessment of tinnitus symptoms and dietary intake, participants were followed at 5 and 10 years. At the 10-year follow-up, lower dietary intake of both iron and zinc was associated with higher tinnitus incidence. Participants in the lowest quintile of iron intake (≤9.51 mg/day) had a 35% higher relative risk of developing tinnitus compared with those with higher intake, while those consuming ≤ 8.48 mg/day of zinc had a 44% higher risk of incident tinnitus. In contrast, interventional evidence for zinc supplementation was not supportive. Person et al. [27] evaluated oral zinc supplementation in tinnitus patients in a review including three randomized controlled trials with a total of 209 participants. Across these trials, zinc supplementation did not produce statistically significant improvements in tinnitus outcomes compared with placebo. No consistent changes were observed in tinnitus loudness, severity, or disability scores, and the pooled analysis showed no therapeutic benefit.

### 3.3. Associations Between BMI, Weight Loss, Physical Activity, and Tinnitus

Evidence concerning BMI, obesity, body composition, and physical activity was derived from population-based, hospital-based, and cross-sectional studies. Gallus et al. [28] provided nationally representative data based on interviews with 2952 adults, in whom anthropometric measurements were used to classify BMI as normal weight, overweight, or obese. Obesity (BMI ≥ 30 kg/m^2^) was significantly associated with a higher likelihood of reporting tinnitus, particularly among participants aged 45 years and older. In multivariable logistic regression, obese individuals had more than twofold higher odds of tinnitus compared with normal-weight participants, whereas overweight individuals showed a weaker, borderline association. The association was stronger when the analysis was restricted to chronic tinnitus lasting at least three months. Hospital-based case–control and observational studies reported similar associations between obesity-related variables and tinnitus. Martines et al. [29] included 46 patients with chronic tinnitus and 74 age- and sex-matched controls, with data collected through medical history, anthropometric assessment, audiometric evaluation, and laboratory testing. Obesity and large neck circumference were significantly more common among tinnitus patients than controls, and the coexistence of obesity and hypertension was associated with markedly higher odds of tinnitus. Torun et al. [30] similarly reported higher mean BMI and greater prevalence of overweight and obesity among 100 patients with chronic subjective tinnitus compared with 113 age- and sex-matched controls. Sogebi et al. [31], in a hospital-based observational study of adult otolaryngology patients, found that obesity was more frequent among patients reporting tinnitus than among controls (21.5% vs. 6.3%; *p* = 0.024). Body composition analyses provided additional insight beyond BMI alone. Han et al. [32] examined 2257 adults, including 204 participants with tinnitus and 2125 without tinnitus. Among men, tinnitus was associated with higher total body fat percentage, greater trunk and leg fat percentage, larger waist circumference, and lower total body fluid and intracellular fluid percentages. In women, these differences were less pronounced and lost significance after adjustment. When obesity was defined using total body fat percentage or waist circumference, tinnitus prevalence was significantly higher among obese men, suggesting that central adiposity and sex-specific body composition patterns may be relevant to the observed association.

Physical activity was examined as a lifestyle-related factor associated with tinnitus outcomes. Chalimourdas et al. [33] analyzed 2751 adults with chronic tinnitus using the International Physical Activity Questionnaire and found that higher levels of moderate-intensity activity were associated with lower tinnitus loudness, whereas vigorous activity was associated with both lower loudness and lower severity. Chen et al. [34], using data from 3826 adults in the U.S. National Health and Nutrition Examination Survey, reported that individuals engaging in physical activity had lower tinnitus prevalence than inactive participants. Subgroup analyses indicated that moderate weekly activity was associated with lower odds of tinnitus, and dose-response modelling suggested that moderate amounts of physical activity showed the most favorable association with tinnitus prevalence. Building on observational associations, three randomized controlled trials examined whether structured lifestyle interventions involving diet, physical activity, and weight reduction were associated with changes in tinnitus-related outcomes. Özbey-Yücel et al. [35] evaluated a 12-week structured weight-loss intervention in 46 obese adults with chronic tinnitus, randomized to diet plus physical activity, diet only, or a control group. Diet plans were individualized by registered dieticians, and participants in the diet plus physical activity group were instructed to achieve at least 10,000 steps per day. Tinnitus severity was assessed using the Tinnitus Handicap Inventory and Visual Analogue Scale, while quality of life was measured using the Short Form Health Survey [39]. Both intervention groups showed significant reductions in body weight and BMI. The diet plus physical activity group achieved a mean weight loss of 6.5 ± 2.6 kg and BMI reduction of 2.1 ± 1.1 kg/m^2^, while the diet-only group achieved a mean weight loss of 4.1 ± 1.0 kg and BMI reduction of 1.5 ± 0.3 kg/m^2^; changes in the control group were negligible. Improvements in tinnitus-related and quality-of-life outcomes were also greater in the intervention groups than in controls. THI scores decreased by −15.0 ± 9.5 points in the diet plus physical activity group and −14.0 ± 10.0 in the diet-only group, compared with −7.0 ± 5.0 in controls (*p* = 0.003). VAS scores declined by −3.0 ± 1.5 and −2.0 ± 1.0 in the intervention groups, respectively, compared with −0.5 ± 0.5 in controls (*p* < 0.01). SF-36 scores improved most in the diet plus physical activity group (+10.0 ± 5.5), followed by the diet-only group (+6.0 ± 2.7) and controls (+3.0 ± 2.0; *p* = 0.001), suggesting that the addition of physical activity may be associated with greater quality-of-life gains. In a subsequent randomized controlled study, the same research group expanded the sample to 63 obese adults with chronic tinnitus and added a physical activity-only group [36]. Participants were allocated to diet plus physical activity, diet only, physical activity only, or control groups for 12 weeks. Body weight decreased significantly in the diet plus physical activity group (−5.9 ± 3.5 kg; *p* < 0.01), diet-only group (−3.4 ± 0.9 kg; *p* < 0.01), and physical activity-only group (−2.0 ± 2.1 kg; *p* < 0.05), while no significant change was observed in controls. Tinnitus-related outcomes also improved in the intervention groups. THI scores decreased by −13.3 ± 2.6 units in the diet plus physical activity group, −9.1 ± 4.5 in the diet-only group, and −8.6 ± 3.8 in the physical activity-only group (*p* < 0.05). VAS severity and annoyance scores were reduced in all intervention groups, with the largest changes observed in the diet plus physical activity group. Participants who lost at least 5% of their initial body weight showed greater reductions in tinnitus severity and VAS scores than those with less than 5% weight loss. Similar findings were reported by Ismail et al. [37] in a randomized controlled trial involving 60 older adults with metabolic syndrome and chronic subjective tinnitus. Participants were allocated to a 12-week lifestyle-modification program combining dietary restriction with supervised treadmill exercise or to a wait-list control group. After the intervention, the lifestyle group showed significant reductions in BMI (32.91 ± 2.23 to 30.76 ± 2.03 kg/m^2^; *p* < 0.001) and waist circumference (110.16 ± 12.98 to 102.26 ± 14.46 cm; *p* < 0.001). Tinnitus-related outcomes also improved, with reductions in VAS severity (6.90 ± 1.38 to 4.74 ± 1.28; *p* < 0.001), VAS discomfort (6.98 ± 1.39 to 4.08 ± 1.06; *p* < 0.001), and THI scores (49.26 ± 9.93 to 33.66 ± 9.09; *p* < 0.001). These post-intervention outcomes were superior to those observed in the wait-list control group, with between-group differences significant for VAS severity, VAS discomfort, and THI (all *p* < 0.001).

## 4. Discussion

This scoping review mapped a heterogeneous body of literature examining relationships between diet, nutritional status, metabolic health, body composition, physical activity, and tinnitus. Overall, the available evidence suggests several recurring associations, particularly for obesity-related factors, lipid metabolism, selected micronutrients, and lifestyle-related variables. However, most included studies were cross-sectional, case–control, or observational in design, and therefore do not allow causal inference. Even where statistically significant associations were reported, these findings should be interpreted cautiously because tinnitus is strongly influenced by age, hearing loss, lifetime noise exposure, psychological distress, cardiovascular disease, diabetes, medication use, and general health status. These factors may confound, mediate, or modify several of the observed associations.

To strengthen the interpretation of the evidence, the included studies should be considered according to both exposure domain and study design. Observational studies primarily identified associations between tinnitus and nutritional, metabolic, anthropometric, or lifestyle-related variables, whereas interventional studies provided limited evidence on changes in tinnitus-related outcomes following structured lifestyle or supplementation approaches. Across domains, the most consistent analytical issue was the difficulty in separating direct associations with tinnitus from indirect pathways involving hearing impairment, cardiometabolic disease, psychological distress, medication use, alcohol consumption, and general health status. Therefore, the present synthesis emphasizes patterns of association, consistency across study types, and methodological limitations rather than causal effects.

Protein and fat intake emerged as relevant dietary factors in the context of tinnitus development and severity, highlighting the potential importance of macronutrient status for auditory health. Population-based and clinical studies suggest that higher protein intake may be associated with lower odds of tinnitus occurrence and symptom severity [14,15], although interventional evidence in this area remains limited. Protein may support cochlear health through its role in tissue repair, immune function, and maintenance of cellular integrity within auditory pathways. This hypothesis is consistent with the possibility that adequate protein intake may be linked to better resilience against degenerative or inflammatory processes associated with tinnitus, although further prospective and interventional studies are needed to clarify the direction and nature of this relationship.

Fat intake and lipid metabolism have also been discussed in relation to auditory outcomes. Diets characterized by higher fat intake or unfavorable lipid profiles have previously been associated with poorer hearing outcomes [40]. The underlying mechanisms remain incompletely understood; however, some studies suggest that elevated blood viscosity and atherosclerotic alterations in cochlear vessels may reduce cochlear blood flow and thereby contribute to auditory dysfunction [41,42,43]. In this context, interventions targeting cholesterol levels, such as low-cholesterol diets and antihyperlipidemic therapy, have shown promising associations with improvements in tinnitus severity in selected patients with hyperlipidemia [17]. Such interventions may also be relevant to broader auditory function, as Rosen et al. [44] observed improved hearing thresholds in individuals following a low-fat diet. These findings are consistent with a potential link between vascular health and auditory function, suggesting that dietary changes may support cochlear health alongside cardiovascular benefits. Findings by Lee HJ et al. [16] further support this association by showing that dyslipidemia-related markers were associated with tinnitus in older adults. Their large-scale cross-sectional analysis demonstrated that individuals with hypertriglyceridemia and elevated total cholesterol/HDL ratios were more likely to report tinnitus. Beyond auditory outcomes, lipid dysregulation may also be linked with the psychological burden associated with tinnitus. Recent findings by Boecking et al. [45] suggest that altered lipid profiles may be associated with greater depressive symptoms in patients with chronic tinnitus, highlighting the potential interaction between metabolic health and emotional distress. Given that tinnitus severity is often modulated by psychological factors, these observations underscore the importance of considering both metabolic and psychosocial dimensions in tinnitus assessment and management.

While macronutrient intake and metabolic health may be linked to tinnitus outcomes through vascular and metabolic pathways, increasing attention has also been directed toward the potential role of micronutrients. Micronutrients are integral to various physiological processes and may be relevant to auditory health. The reviewed studies indicate that selected nutritional factors, including vitamins, antioxidants, and minerals, may be associated with tinnitus prevalence and severity. Vitamin B2 (riboflavin), B3 (niacin), and B12 (cobalamin) are part of the B-complex group, each contributing to cellular energy production and nerve function, both of which are important for auditory system integrity. Vitamin B2 may be relevant to tinnitus through its role in mitochondrial energy production and oxidation-reduction reactions. Its potential relationship with oxidative stress is particularly relevant, as oxidative damage has been proposed as one of the mechanisms involved in the pathophysiology of tinnitus [46]. Historically, vitamin B3 has been used in tinnitus management [47]. Although the precise mechanism underlying its association with tinnitus remains uncertain, it has been hypothesized that the vasodilatory properties of vitamin B3 may be relevant to auditory symptoms. For vitamins B2 and B3, results from the KNHANES survey by Lee DY et al. [18] demonstrated that lower intake was consistently associated with higher tinnitus prevalence and greater symptom annoyance. Although the observed effect size was modest (OR ≈ 1.25 for lower B2 intake), the consistency of this association across multiple age groups suggests that these vitamins may be relevant to auditory health. In contrast, evidence on vitamin B12 remains less consistent. Berkiten et al. [19] reported high rates of deficiency among tinnitus patients but found no significant difference compared with controls, suggesting that deficiency may also be common in the general population. Interventional evidence remains limited; however, a randomized controlled trial by Singh et al. [20] demonstrated that vitamin B12 supplementation was associated with reductions in tinnitus severity among patients with confirmed deficiency. These findings suggest that while micronutrient status may be relevant in certain patient subgroups, the potential clinical value of supplementation likely depends on baseline nutritional status. Among individual micronutrients, vitamin D has also been investigated in relation to tinnitus. Evidence from both clinical and population-based studies suggests that vitamin D deficiency may be associated with increased tinnitus prevalence and symptom severity. In a case–control study, Nowaczewska et al. [21] reported significantly lower serum 25(OH)D levels in patients with chronic tinnitus compared with controls, with deficiency associated with higher THI and VAS scores. Similar trends were observed in analyses based on the Korea National Health and Nutrition Examination Survey, where individuals in the lowest vitamin D quartile showed higher odds of reporting tinnitus than those with higher vitamin D levels [22].

In addition to vitamins, antioxidant compounds have also been explored in relation to tinnitus management. However, the available evidence remains heterogeneous, reflecting the wide variety of compounds tested, as well as differences in study design, intervention duration, and patient populations. Some clinical studies have reported favorable changes in tinnitus-related outcomes following antioxidant supplementation. For example, Petridou et al. [23] observed reductions in tinnitus loudness and improvements in patient-reported outcomes following treatment with a multivitamin-multimineral preparation combined with α-lipoic acid. Similarly, Savastano et al. [24] reported decreases in oxidative stress markers accompanied by reductions in tinnitus loudness and subjective annoyance after antioxidant therapy. These findings are consistent with the hypothesis that oxidative stress may be involved in tinnitus pathophysiology and that antioxidant-based approaches may be relevant in selected patients. Nevertheless, not all studies have demonstrated favorable outcomes. In a randomized, double-blind, placebo-controlled trial, Polanski et al. [25] evaluated several antioxidant regimens, including Ginkgo biloba, α-lipoic acid with vitamin C, and papaverine with vitamin E, and found no significant improvements in tinnitus severity compared with placebo. Such discrepancies may reflect differences in the composition and dosage of antioxidant formulations, treatment duration, baseline oxidative status, hearing-loss characteristics, comorbidities, and other patient-specific factors. Overall, although antioxidant therapy remains biologically plausible through its relationship with oxidative stress, current evidence is heterogeneous and does not yet support its routine use in tinnitus management.

Beyond vitamins and antioxidants, several studies have examined the potential relevance of trace elements, particularly zinc and iron, in relation to tinnitus. These micronutrients are involved in key physiological processes relevant to auditory function. Iron plays a central role in oxygen transport and cellular energy metabolism, while zinc contributes to cochlear physiology and synaptic activity within auditory pathways [48]. Observational evidence suggests that lower intake or deficiency of these minerals may be associated with tinnitus occurrence. In a 10-year prospective cohort study, Tang et al. [26] reported that lower dietary intake of zinc and iron was associated with a higher incidence of tinnitus. Such findings are consistent with the hypothesis that adequate mineral intake may be relevant to maintaining auditory system function. However, interventional evidence has not consistently demonstrated therapeutic benefits. In the study by Person et al. [27], the authors found that zinc supplementation did not significantly improve tinnitus loudness, severity, or disability compared with placebo. These findings suggest that although low intake of certain trace elements may be associated with tinnitus occurrence or incidence, current clinical evidence does not support routine mineral supplementation as an effective treatment strategy.

Body Mass Index (BMI) is closely linked to diet, since diet and nutrient intake play a key role in determining body weight. Across diverse study designs and populations, obesity was consistently associated with tinnitus across several observational studies. Large-scale surveys and hospital-based studies alike have shown that individuals with elevated BMI are more likely to report both incident and chronic tinnitus compared with their normal-weight counterparts [28,29,30,31]. These associations appear robust, with investigations also highlighting the amplifying role of comorbid conditions such as hypertension [29]. More recent analyses that considered body composition have further refined this picture, suggesting that central adiposity and altered fat distribution, rather than overall weight alone, may be particularly relevant [32]. Collectively, these findings indicate a potential association between obesity, metabolic disturbances, and tinnitus risk. Beyond risk, interventional evidence suggests that weight reduction may also influence tinnitus severity. Randomized trials by Özbey-Yücel et al. [35,36] demonstrated that lifestyle programs incorporating dietary modification and physical activity were associated with improvements in tinnitus severity and quality-of-life measures. Similar findings were reported by Ismail et al. [37], who observed reductions in BMI and waist circumference alongside improvements in tinnitus-related outcomes following a structured lifestyle intervention in older adults with metabolic syndrome. Taken together, observational and clinical evidence suggest that obesity might be both a risk factor and a modifiable therapeutic target in tinnitus. While the observational studies are limited by potential confounding, the convergence of results across settings and the replication of symptom improvements in randomized interventional studies indicate that weight reduction may be associated with improvements in tinnitus severity and quality of life.

A particularly important issue in interpreting these findings is the role of hearing impairment. Hearing loss is one of the strongest and most consistently reported factors associated with tinnitus, and noise exposure is a major determinant of both auditory threshold shifts and tinnitus symptoms [49,50,51]. Therefore, hearing status may confound, mediate, or modify the associations observed between nutritional, metabolic, lifestyle-related factors, and tinnitus. This is especially relevant because obesity, dyslipidemia, diabetes, cardiovascular disease, and other metabolic disturbances may also be associated with poorer auditory function. Consequently, an apparent association between BMI, lipid profile, diet, physical activity, or micronutrient status and tinnitus may partly reflect their relationship with underlying hearing impairment rather than an independent association with tinnitus itself. Some of the included studies adjusted for hearing-related variables, including hearing loss or noise exposure, whereas others relied primarily on self-reported tinnitus outcomes without detailed audiometric adjustment. This heterogeneity limits direct comparison across studies and means that the independent relationship between nutritional or metabolic factors and tinnitus remains uncertain.

### 4.1. Limitations

The studies included in this review provide valuable insight into the potential relationships between dietary factors, BMI, physical activity, metabolic health, and tinnitus. However, several limitations should be considered when interpreting the findings. First, the generalizability of the evidence is limited because several studies were conducted in specific geographic, ethnic, or clinical populations. For example, multiple analyses relied on data from the Korea National Health and Nutrition Examination Survey (KNHANES), which provides robust population-based data but remains specific to the Korean population. Second, exposure assessment varied considerably across studies and often relied on self-reported dietary or lifestyle measures, including food-frequency questionnaires and physical-activity questionnaires. Although such tools are commonly used in epidemiological research, they may introduce recall bias, reporting bias, and measurement error. Third, most of the available evidence was derived from cross-sectional, case–control, or other observational designs. Consequently, the observed associations should not be interpreted as evidence of causality, and residual confounding or reverse causality cannot be excluded. This is particularly important in relation to hearing impairment. Hearing loss and noise exposure are among the strongest factors associated with tinnitus, yet not all included studies incorporated audiometric thresholds, detailed noise-exposure history, or hearing-related covariates in multivariable models. Because metabolic, nutritional, and lifestyle-related factors may also be associated with hearing impairment, the observed associations between diet, BMI, physical activity, metabolic health, and tinnitus cannot be assumed to be independent of hearing function unless adequately adjusted for audiometric status and noise exposure. Finally, because this was a scoping review, no formal risk-of-bias assessment or quantitative meta-analysis was performed. The included studies differed substantially in design, population characteristics, exposure definitions, tinnitus outcome measures, and adjustment strategies, which limited direct comparability between studies. Therefore, the present review should be interpreted as a structured evidence map rather than as evidence of definitive causal or therapeutic effects.

### 4.2. Future Research

Future research should address the current predominance of observational evidence by conducting large, well-designed randomized controlled trials to test whether dietary modification, weight reduction, and physical activity can reduce tinnitus severity. Standardization of outcome measures, including both validated questionnaires and objective audiological tests, is essential to enable synthesis across studies. More diverse populations should be included, as most existing evidence comes from restricted geographic or clinical settings.

## 5. Conclusions

The available literature suggests potential associations between dietary factors, micronutrient status, obesity, physical activity, and tinnitus occurrence or symptom severity. Although the evidence is heterogeneous and largely based on observational studies, several reports indicate that metabolic health and lifestyle behaviors may be associated with tinnitus-related outcomes. Interventional evidence further suggests that lifestyle-based approaches, including dietary modification and increased physical activity, may be associated with improvements in symptom burden and quality of life in selected patient groups. However, the current evidence base remains constrained by methodological variability, potential confounding, and reliance on self-reported dietary or lifestyle assessments. Further well-designed prospective studies and randomized controlled trials are required to clarify these relationships and determine the potential role of lifestyle interventions in tinnitus management. Nonetheless, the findings highlight metabolic and lifestyle-related factors as relevant emerging areas of interest in tinnitus research.

## Figures and Tables

**Table 1:** Characteristics and main findings of included studies.

Study	Design	Population	N	Exposure/Factor	Tinnitus Definition/Assessment	Adjustment Variables	Key Findings	Key Limitations
Dawes et al., [14]	Cross-sectional	UK Biobank adults	34,576	Protein intake	Self-reported tinnitus	Demographic, lifestyle, and health-related factors	Higher protein intake associated with lower odds of tinnitus	Cross-sectional design; dietary self-report; residual confounding possible
Jarach et al., [15]	Case–control	Hospital-based sample, Italy; tinnitus vs. controls	383	Diet; protein-rich foods	Idiopathic tinnitus case status	Sex, age, education, BMI, smoking, alcohol consumption, and hearing loss	Higher poultry, prosciutto, and legume intake associated with lower odds of tinnitus	Case–control design; recall bias; hospital-based controls
Lee HJ et al., [16]	Cross-sectional	KNHANES; adults ≥ 60 y	6021	Lipids; TG, TC/HDL-C	Self-reported tinnitus prevalence and annoyance severity	Age, sex, hypertension, diabetes, dyslipidemia, smoking, obesity, noise exposure, psychological factors	Hypertriglyceridemia and high TC/HDL-C associated with tinnitus and severe annoyance	Cross-sectional design; self-reported tinnitus; residual confounding possible
Sutbas et al., [17]	Interventional, non-RCT	Men with hyperlipidemia and tinnitus	42	Low-cholesterol diet ± statin therapy	Tinnitus rating 1–10 and tinnitus questionnaire	Limited/not clearly multivariable adjusted	Lipid responders showed lower tinnitus scores; non-responders showed no comparable benefit	Small sample; male-only; no randomized control group
Lee DY et al., [18]	Cross-sectional	KNHANES	7621	Vitamin B2/B3 intake	Self-reported tinnitus prevalence and annoyance	Multivariable nutritional analysis; covariates as reported	Lower B2 associated with higher prevalence; lower B3 associated with greater annoyance	Cross-sectional design; dietary self-report; residual confounding possible
Berkiten et al., [19]	Case–control + replacement	Non-pulsatile tinnitus vs. controls	120	Vitamin B12 status; IM B12 in deficient patients	Non-pulsatile tinnitus; VAS	Limited/not clearly adjusted	B12 deficiency common in both groups; replacement associated with minimal non-significant VAS change overall	Small control group; uncontrolled replacement phase
Singh et al., [20]	RCT, placebo-controlled	Chronic tinnitus patients	40	IM methylcobalamin	Chronic tinnitus; TSI and VAS	Randomization; subgroup by B12 deficiency	Improvement observed only in B12-deficient treatment subgroup	Small sample; short follow-up; subgroup findings
Nowaczewska et al., 2021 [21]	Case–control	Chronic subjective tinnitus vs. controls	300	Serum 25(OH)D	Chronic subjective tinnitus; THI and VAS	Not clearly reported	Lower vitamin D in tinnitus; deficiency associated with higher THI/VAS	Case–control design; no intervention; lifestyle, sun/noise exposure, physical activity and diet not fully accounted for
Aliyeva et al., [22]	Cross-sectional	KNHANES adults	16,408	Vitamin D quartiles	Self-reported tinnitus prevalence	Demographic and lifestyle factors	Lowest vitamin D quartile associated with higher tinnitus odds	Cross-sectional design; residual confounding possible
Petridou et al., [23]	RCT, double-blind placebo-controlled	Tinnitus ≥ 6 months	63	Multivitamin-multimineral + α-lipoic acid	Tinnitus loudness, MML, THI, VAS, TFI subscales	Randomization	Active arm showed lower loudness/MML and improved patient-reported outcomes	Modest sample; multi-compound intervention; limited attribution to individual nutrients
Savastano et al., [24]	Pre-post trial	Unilateral idiopathic tinnitus	31	Antioxidant regimen	Tinnitus loudness, VAS; ROS markers	No placebo control/limited adjustment	Lower loudness and VAS; lower MDA/4-HNE after treatment	Small sample; uncontrolled design; short-term follow-up
Polanski et al., [25]	RCT, double-blind placebo-controlled	≥60 y; tinnitus with SNHL	58	Ginkgo/ALA + VitC/papaverine + VitE	Chronic tinnitus with SNHL; THI	Randomization	No significant benefit vs. placebo	Older SNHL population; multiple regimens; limited power for subgroup effects
Tang et al., [26]	Prospective cohort	Adults ≥ 50 y	2947	Dietary iron and zinc intake	Incident tinnitus over 10 years	Age, sex, dizziness symptoms, middle ear infections, and hearing loss	Lowest iron and zinc intake associated with higher incident tinnitus	Dietary self-report; residual confounding possible
Person et al., [27]	Systematic review	Tinnitus patients in RCTs	209	Zinc supplementation	Tinnitus loudness, severity, disability	Not applicable	No consistent benefit vs. placebo	Limited number and quality of included trials
Gallus et al., [28]	Cross-sectional	National sample, Italy	2952	BMI categories	Self-reported tinnitus, including chronic tinnitus	Demographic/lifestyle variables, as reported	Obesity associated with higher tinnitus odds, stronger for chronic tinnitus	Cross-sectional design; self-reported tinnitus; residual confounding possible
Martines et al., [29]	Case–control	ENT clinic; tinnitus vs. controls	120	BMI and metabolic factors, including hypertension	Chronic tinnitus; audiometric evaluation performed	Age/sex matching; clinical and metabolic variables	Obesity and large neck circumference more common; obesity + hypertension associated with markedly higher odds	Small sample; case–control design; residual confounding possible
Torun et al., [30]	Case–control	Chronic tinnitus vs. controls	213	BMI	Chronic subjective tinnitus	Age/sex matching	Higher BMI and more frequent overweight/obesity in tinnitus patients	Case–control design; limited adjustment
Sogebi et al., [31]	Cross-sectional observational	ENT clinic patients	NR	BMI/obesity	Self-reported tinnitus symptoms; PTA performed	Limited/not clearly adjusted	Obesity more common among tinnitus patients than controls	Clinic-based sample; selection bias; limited adjustment
Han et al., [32]	Cross-sectional	KNHANES	2257	Body composition; fat %, waist circumference	Self-reported tinnitus	Demographic/clinical variables, as reported	Central adiposity associated with tinnitus, particularly in men	Cross-sectional design; sex-specific findings; residual confounding possible
Chalimourdas et al., [33]	Cross-sectional	Adults with chronic tinnitus	2751	Physical activity, IPAQ	Chronic tinnitus; loudness and severity	Covariates as reported in the original study	Higher activity associated with lower loudness and severity	Cross-sectional design; self-reported activity; residual confounding possible
Chen et al., [34]	Cross-sectional	NHANES adults	3826	Physical activity, minutes/week	Self-reported tinnitus prevalence	Demographic and health covariates, as reported	Any PA associated with lower prevalence; moderate PA showed most favorable association	Cross-sectional design; self-reported exposure/outcome; residual confounding possible
Özbey-Yücel et al., [35]	RCT	Obese tinnitus patients	46	Diet vs. diet + PA vs. control	Chronic tinnitus; THI, VAS; SF-36	Randomization	Lifestyle arms showed lower THI/VAS and higher QoL vs. control	Small sample; short duration; selected obese population
Özbey-Yücel et al., [36]	RCT	Obese tinnitus patients	63	Diet vs. PA vs diet + PA vs. control	Chronic tinnitus; THI and VAS	Randomization	All active arms improved; combined diet + PA showed greatest changes	Small groups; short follow-up; selected obese population
Ismail et al., [37]	RCT	≥65 y; metabolic syndrome and tinnitus	60	Dietary restriction + treadmill exercise	Chronic subjective tinnitus; THI, VAS severity/discomfort	Randomization	Significant improvements vs. control	Selected metabolic syndrome population; short duration

Abbreviations: ALA, α-lipoic acid; BMI, body mass index; HDL-C, high-density lipoprotein cholesterol; IM, intramuscular; IPAQ, International Physical Activity Questionnaire; KNHANES, Korea National Health and Nutrition Examination Survey; MDA, malondialdehyde; MML, minimum masking level; NR, not reported; PA, physical activity; RCT, randomized controlled trial; ROS, reactive oxygen species; SF-36, Short Form Health Survey; SNHL, sensorineural hearing loss; TC, total cholesterol; TG, triglycerides; THI, Tinnitus Handicap Inventory; TFI, Tinnitus Functional Index; TSI, Tinnitus Severity Index; VAS, Visual Analogue Scale.

## Data Availability

No new data were created in this study. This scoping review was based exclusively on data extracted from previously published articles. Data sharing is not applicable to this article.

## Supplementary Materials

The following supporting information can be downloaded at: https://www.mdpi.com/article/10.3390/jcm15114274/s1, Table S1. Database-specific search strings. Table S2. Preferred Reporting Items for Systematic reviews and Meta-Analyses extension for Scoping Reviews (PRISMA-ScR) Checklist.

## References

[1] Baguley D., McFerran D., Hall D. (2013). Tinnitus. Lancet.

[2] Skarżyński H., Rogowski M., Fabijańska A., Bartnik G., Raj-Koziak D., Jahnke K., Fischer M. (2000). The Epidemiology of Hearing Disorders in Poland. Proceedings of the 4th European Congress of Oto-Rhino-Laryngology Head and Neck Surgery, Berlin, Germany, 13–18 May 2000.

[3] Raj-Koziak D., Gos E., Świerniak W., Skarżyński H., Skarżyński P.H. (2021). Prevalence of Tinnitus in a Sample of 43,064 Children in Warsaw, Poland. Int. J. Audiol..

[4] Henton A., Tzounopoulos T. (2021). What’s the Buzz? The Neuroscience and the Treatment of Tinnitus. Physiol. Rev..

[5] Perez-Carpena P., Lopez-Escamez J.A., Gallego-Martinez Á. (2024). A Systematic Review on the Genetic Contribution to Tinnitus. J. Assoc. Res. Otolaryngol..

[6] Świerniak W., Gos E., Skarżyński P.H., Czajka N., Skarżyński H. (2020). Personal Music Player Use and Other Noise Hazards among Children 11 to 12 Years Old. Int. J. Environ. Res. Public Health.

[7] Mennink L.M., Aalbers M.W., van Dijk P., van Dijk J.M.C. (2022). The Role of Inflammation in Tinnitus: A Systematic Review and Meta-Analysis. J. Clin. Med..

[8] Chmiela S., Skarżyński P.H., Raj-Koziak D. (2025). Znaczenie BMI i redukcji masy ciała w prewencji oraz terapii szumów usznych: Przegląd literatury. Nowa Audiofonologia.

[9] Newman C.W., Jacobson G.P., Spitzer J.B. (1996). Development of the Tinnitus Handicap Inventory. Arch. Otolaryngol. Head Neck Surg..

[10] Meikle M.B., Henry J.A., Griest S.E., Stewart B.J., Abrams H.B., McArdle R., Myers P.J., Newman C.W., Sandridge S., Turk D.C. (2012). The Tinnitus Functional Index: Development of a New Clinical Measure for Chronic, Intrusive Tinnitus. Ear Hear..

[11] Skarżyński H., Gos E., Raj-Koziak D., Skarżyński P.H. (2018). Skarżyński Tinnitus Scale: Validation of a Brief and Robust Tool for Assessing Tinnitus in a Clinical Population. Eur. J. Med. Res..

[12] Fuller T., Cima R., Langguth B., Mazurek B., Vlaeyen J.W.S., Hoare D.J. (2020). Cognitive Behavioural Therapy for Tinnitus. Cochrane Database Syst. Rev..

[13] Tricco A.C., Lillie E., Zarin W., O’Brien K.K., Colquhoun H., Levac D., Moher D., Peters M.D.J., Horsley T., Weeks L. (2018). PRISMA Extension for Scoping Reviews (PRISMA-ScR): Checklist and Explanation. Ann. Intern. Med..

[14] Dawes P., Cruickshanks K.J., Marsden A., Moore D.R., Munro K.J. (2020). Relationship Between Diet, Tinnitus, and Hearing Difficulties. Ear Hear..

[15] Jarach C.M., Lugo A., Garavello W., van den Brandt P.A., Odone A., Cederroth C.R., Bosetti C., Gallus S. (2023). The Role of Diet in Tinnitus Onset: A Hospital-Based Case-Control Study from Italy. Nutrients.

[16] Lee H.J., Lee D.C., Kim C.O. (2024). The Association Between Serum Lipid Levels and Tinnitus Prevalence and Severity in Korean Elderly: A Nationwide Population-Based Cross-Sectional Study. Yonsei Med. J..

[17] Sutbas A., Yetiser S., Satar B., Akcam T., Karahatay S., Saglam K. (2007). Low-Cholesterol Diet and Antilipid Therapy in Managing Tinnitus and Hearing Loss in Patients with Noise-Induced Hearing Loss and Hyperlipidemia. Int. Tinnitus J..

[18] Lee D.Y., Kim Y.H. (2018). Relationship Between Diet and Tinnitus: Korea National Health and Nutrition Examination Survey. Clin. Exp. Otorhinolaryngol..

[19] Berkiten G., Kumral T.L., Saltürk Z., Yildirim G., Atar Y., Uyar Y. (2013). Vitamin B12 Levels in Patients with Tinnitus and Effectiveness of Vitamin B12 Treatment on Tinnitus. J. Laryngol. Otol..

[20] Singh C., Kawatra R., Gupta J. (2016). Therapeutic Role of Vitamin B12 in Patients of Chronic Tinnitus: A Pilot Study. Noise Health.

[21] Nowaczewska M., Wrzosek M., Wrzosek P., Wojciak R.W. (2021). The Role of Vitamin D in Subjective Tinnitus—A Case-Control Study. PLoS ONE.

[22] Aliyeva A., Han J.S., Kim Y., Lim J.H., Seo J.H., Park S.N. (2024). Vitamin D Deficiency as a Risk Factor of Tinnitus: An Epidemiological Study. Ann. Otol. Rhinol. Laryngol..

[23] Petridou A.I., Zagora E.T., Petridis P., Korres G.S., Gazouli M., Xenelis I., Kyrodimos E., Kontothanasi G., Kaliora A.C. (2019). The Effect of Antioxidant Supplementation in Patients with Tinnitus and Normal Hearing or Hearing Loss: A Randomized, Double-Blind, Placebo Controlled Trial. Nutrients.

[24] Savastano M., Brescia G., Marioni G. (2007). Antioxidant Therapy in Idiopathic Tinnitus: Preliminary Outcomes. Arch. Med. Res..

[25] Polanski J.F., Soares A.D., de Mendonça Cruz O.L. (2016). Antioxidant Therapy in the Elderly with Tinnitus. Braz. J. Otorhinolaryngol..

[26] Tang D., Shekhawat G.S., Burlutsky G., Mitchell P., Gopinath B. (2024). The Association between Dietary Intakes of Vitamins and Minerals with Tinnitus. Nutrients.

[27] Person O.C., Puga M.E., da Silva E.M., Torloni M.R. (2016). Zinc Supplementation for Tinnitus. Cochrane Database Syst. Rev..

[28] Gallus S., Lugo A., Garavello W., Bosetti C., Santoro E., Colombo P., Perin P., La Vecchia C., Langguth B. (2015). Prevalence and Determinants of Tinnitus in the Italian Adult Population. Neuroepidemiology.

[29] Martines F., Sireci F., Cannizzaro E., Costanzo R., Martines E., Mucia M., Plescia F., Salvago P. (2015). Clinical Observations and Risk Factors for Tinnitus in a Sicilian Cohort. Eur. Arch. Otorhinolaryngol..

[30] Torun M.T., Yildirim E., Dagli S., Ozkan M., Demirci S. (2016). Do Body Mass Index and Demographic Data Affect Subjective Tinnitus?. Int. J. Clin. Exp. Med..

[31] Sogebi O.A. (2013). Characterization of Tinnitus in Nigeria. Auris Nasus Larynx.

[32] Han S.Y., Lee S.Y., Suh M.W., Lee J.H., Park M.K. (2024). Associations between Tinnitus and Body Composition: A Cross-Sectional Study. Sci. Rep..

[33] Chalimourdas A., Hansen D., Verboven K., Michiels S. (2025). The Relationship between Physical Activity and Tinnitus Loudness and Severity: A Cross-Sectional Study. Ear Hear..

[34] Chen S., Yang X., Jiang Y., Wu F., Li Y., Qiu J., Tong B., Liu Y. (2023). Associations between Physical Activity, Tinnitus, and Tinnitus Severity. Ear Hear..

[35] Özbey-Yücel Ü., Aydoğan Z., Tokgöz-Yilmaz S., Uçar A., Ocak E., Beton S. (2021). The Effects of Diet and Physical Activity Induced Weight Loss on the Severity of Tinnitus and Quality of Life: A Randomized Controlled Trial. Clin. Nutr. ESPEN.

[36] Özbey-Yücel Ü., Uçar A., Aydoğan Z., Tokgöz-Yilmaz S., Beton S. (2023). The Effects of Dietary and Physical Activity Interventions on Tinnitus Symptoms: An RCT. Auris Nasus Larynx.

[37] Ismail A.M.A., Tolba A.M.N. (2025). Effectiveness of Lifestyle-Modification Approach (a Randomized-Controlled Program of Diet Restriction and Treadmill Walking Exercise) on Elderly’s Metabolic Syndrome-Associated Subjective Tinnitus. Eur. Arch. Otorhinolaryngol..

[38] Neri S., Mauceri B., Cilio D., Bordonaro F., Messina A., Malaguarnera M., Savastano M., Brescia G., Manci S., Celadini M. (2002). Tinnitus and Oxidative Stress in a Selected Series of Elderly Patients. Arch. Gerontol. Geriatr. Suppl..

[39] Brazier J.E., Harper R., Jones N.M., O’Cathain A., Thomas K.J., Usherwood T., Westlake L. (1992). Validating the SF-36 Health Survey Questionnaire: New Outcome Measure for Primary Care. BMJ.

[40] Spankovich C., Le Prell C.G. (2014). Associations between Dietary Quality, Noise, and Hearing: Data from the National Health and Nutrition Examination Survey, 1999–2002. Int. J. Audiol..

[41] Hildesheimer M., Rubinstein M., Nuttal A.L., Lawrence M. (1982). Influence of Blood Viscosity on Cochlear Action Potentials and Oxygenation. Hear. Res..

[42] Suzuki K., Kaneko M., Murai K. (2000). Influence of Serum Lipids on Auditory Function. Laryngoscope.

[43] Cunningham D.R., Goetzinger C.P. (1974). Extra-High Frequency Hearing Loss and Hyperlipidemia. Int. J. Audiol..

[44] Rosen S., Olin P., Rosen H.V. (1970). Dietary Prevention of Hearing Loss. Acta Otolaryngol..

[45] Boecking B., Klasing S., Brueggemann P., Rose M., Mazurek B. (2024). Lipid Parameters and Depression in Patients with Chronic Tinnitus: A Cross-Sectional Observation. J. Psychosom. Res..

[46] Neri S., Signorelli S., Pulvirenti D., Mauceri B., Cilio D., Bordonaro F., Abate G., Interlandi D., Misseri M., Ignaccolo L. (2006). Oxidative Stress, Nitric Oxide, Endothelial Dysfunction and Tinnitus. Free Radic. Res..

[47] Hulshof J.H., Vermeij P. (1987). The Effect of Nicotinamide on Tinnitus: A Double-Blind Controlled Study. Clin. Otolaryngol..

[48] Schieffer K.M., Connor J.R., Pawelczyk J.A., Sekhar D.L. (2017). The Relationship between Iron Deficiency Anemia and Sensorineural Hearing Loss in the Pediatric and Adolescent Population. Am. J. Audiol..

[49] Biswas R., Genitsaridi E., Trpchevska N., Lugo A., Schlee W., Cederroth C.R., Gallus S., Hall D.A. (2023). Low Evidence for Tinnitus Risk Factors: A Systematic Review and Meta-Analysis. J. Assoc. Res. Otolaryngol..

[50] Kim H.J., Lee H.J., An S.Y., Sim S., Park B., Kim S.W., Lee J.S., Hong S.K., Choi H.G. (2015). Analysis of the Prevalence and Associated Risk Factors of Tinnitus in Adults. PLoS ONE.

[51] Hobeika L., Fillingim M., Tanguay-Sabourin C., Roy M., Londero A., Samson S., Vachon-Presseau E. (2025). Tinnitus Risk Factors and Its Evolution over Time. Nat. Commun..

