# Supplementary material for: Associations of Dietary Factors, Body Mass Index, and Physical Activity with Tinnitus: A Scoping Review
Source: J Clin Med. 2026 Jun 1;15(11):4274. doi: 10.3390/jcm15114274 (PMC13258421; doi:10.3390/jcm15114274)
Supplement: Supplementary file 1 [file jcm-15-04274-s001.zip › Supplementary Table S1. Database-specific search strings. .pdf]

Database	Search string
PubMed	(tinnitus) AND (diet OR nutrition OR antioxidants OR vitamins OR minerals OR macronutrients OR micronutrients OR BMI OR "body mass index" OR obesity OR "weight loss" OR "weight reduction" OR "physical activity" OR exercise)
Web of Science	TS=(tinnitus) AND TS=(diet OR nutrition OR antioxidants OR vitamins OR minerals OR macronutrients OR micronutrients OR BMI OR "body mass index" OR obesity OR "weight loss" OR "weight reduction" OR "physical activity" OR exercise)
Cochrane Library	(tinnitus):ti,ab,kw AND (diet OR nutrition OR antioxidants OR vitamins OR minerals OR macronutrients OR micronutrients OR BMI OR "body mass index" OR obesity OR "weight loss" OR "weight reduction" OR "physical activity" OR exercise):ti,ab,kw

Supplementary Table S1. Database-specific search strings.
